# 5-Chloro-2-nitro­phenol

**DOI:** 10.1107/S1600536812014638

**Published:** 2012-04-13

**Authors:** Dong-mei Ren

**Affiliations:** a Security and Environment Engineering College, Capital University of Economics and Business, Beijing 10070, People’s Republic of China

## Abstract

The asymmetric unit of the title compound, C_6_H_4_ClNO_3_, contains two independent mol­ecules in which the dihedral angles between the benzene ring and the nitro groups are 2.5 (1) and 8.5 (1)°. Intra­molecular O—H⋯O hydrogen bonds involving the hy­droxy and nitro substituents result in the formation of *S*(6) six-membered rings. In the crystal, O—H⋯O, O—H⋯Cl and C—H⋯O hydrogen bonds together with Cl⋯O contacts [3.238 (3) and 3.207 (3) Å] generate a three-dimensional network.

## Related literature

For background to applications of the title compound and its synthesis, see: Richard (1971 ▶). For bond-length data, see: Allen *et al.* (1987 ▶) and for hydrogen-bond motifs, see: Bernstein *et al.* (1995 ▶).
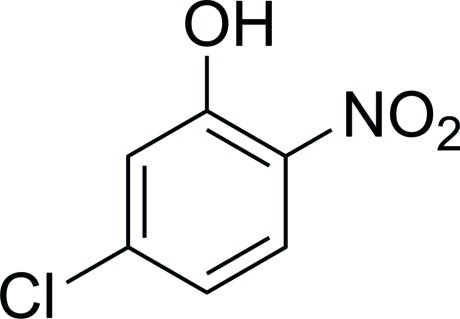


## Experimental

### 

#### Crystal data

C_6_H_4_ClNO_3_
*M* *_r_* = 173.55
Triclinic, 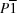

*a* = 7.5390 (15) Å
*b* = 8.1640 (16) Å
*c* = 13.132 (3) Å
α = 94.75 (3)°
β = 96.48 (3)°
γ = 116.46 (3)°
*V* = 710.9 (2) Å^3^
*Z* = 4
Mo *K*α radiation
μ = 0.49 mm^−1^
*T* = 293 K
0.20 × 0.10 × 0.10 mm

#### Data collection

Enraf–Nonius CAD-4 diffractometer
Absorption correction: ψ scan (North *et al.*, 1968 ▶) *T*_min_ = 0.909, *T*_max_ = 0.953
2808 measured reflections
2596 independent reflections
1833 reflections with *I* > 2σ(*I*)
*R*_int_ = 0.075
3 standard reflections every 200 reflections intensity decay: 1%

#### Refinement

*R*[*F* ^2^ > 2σ(*F* ^2^)] = 0.058
*wR*(*F* ^2^) = 0.171
*S* = 1.01
2596 reflections
200 parameters
H-atom parameters constrained
Δρ_max_ = 0.32 e Å^−3^
Δρ_min_ = −0.36 e Å^−3^

### 

Data collection: *CAD-4 Software* (Enraf–Nonius, 1985 ▶); cell refinement: *CAD-4 Software*; data reduction: *XCAD4* (Harms & Wocadlo, 1995 ▶); program(s) used to solve structure: *SHELXS97* (Sheldrick, 2008 ▶); program(s) used to refine structure: *SHELXL97* (Sheldrick, 2008 ▶); molecular graphics: *SHELXTL* (Sheldrick, 2008 ▶); software used to prepare material for publication: *SHELXTL*.

## Supplementary Material

Crystal structure: contains datablock(s) I, global. DOI: 10.1107/S1600536812014638/sj5233sup1.cif

Structure factors: contains datablock(s) I. DOI: 10.1107/S1600536812014638/sj5233Isup2.hkl

Supplementary material file. DOI: 10.1107/S1600536812014638/sj5233Isup3.cml

Additional supplementary materials:  crystallographic information; 3D view; checkCIF report

## Figures and Tables

**Table 1:** Hydrogen-bond geometry (Å, °)

*D*—H⋯*A*	*D*—H	H⋯*A*	*D*⋯*A*	*D*—H⋯*A*
O3—H3*A*⋯O2	0.82	1.91	2.605 (5)	142
O6—H6*A*⋯O4	0.82	1.88	2.581 (4)	143
O3—H3*A*⋯O6^i^	0.82	2.71	3.350 (5)	136
O6—H6*A*⋯Cl2^ii^	0.82	2.70	3.207 (3)	121
C2—H2*A*⋯O5^iii^	0.93	2.49	3.155 (5)	129

Symmetry codes: (i) 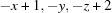; (ii) 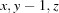; (iii) 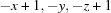.

**Fig. 1.**
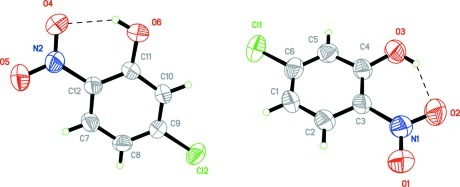
The molecular structure of (I), with the atom-numbering scheme. Displacement ellipsoids are drawn at the 50% probability level and intramolecular hydrogen bonds are drawn as dashed lines.

**Fig. 2.**
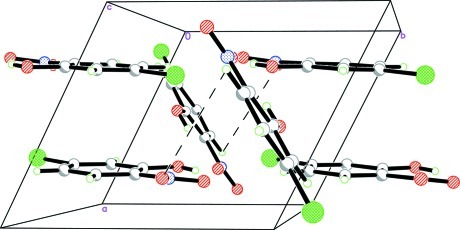
A packing diagram for (I) with hydrogen bonds drawn as dashed lines.

## supplementary crystallographic information

### Comment

The title compound, 5-chloro-2-nitrophenol, is an important intermediate in the preparation of commercially important materials such as lamprecides, agricultural chemicals and dyestuffs (Richard, 1971). We report here the crystal structure of the title compound, (I).

The asymmetric unit contains two molecules of 5-chloro-2-nitrophenol, Fig. 1. The dihedral angles between the C1—C6 ring plane and that of the nitro group N1/O1/O2 is 2.5 (1)° while that between the C7—C12 plane and that of N2/04/05 is 8.5 (1)°. Intramolecular O3—H3A···O2 and O6—H6A···O4 hydrogen bonds form S(6) rings in both molecules (Bernstein *et al.*, 1995). Bond distances in both molecules are normal (Allen *et al.* 1987).

In the crystal structure intermolecular O—H···O, O—H···Cl, and C—H···O hydrogen bonds, Table 1, together with and Cl2···O2 and Cl2···O6 contacts with distances 3.238 (3) and 3.207 (3) Å respectively generate a three dimensional network structure, Fig 2.

### Experimental

The title compound, (I) was prepared by a method reported in literature (Richard, 1971). Crystals were obtained by dissolving (I) (0.1 g) in methanol (30 ml) and evaporating the solvent slowly at room temperature for about 8 d.

### Refinement

All H atoms were positioned geometrically and constrained to ride on their parent atoms, with C—H = 0.93 Å for aromatic H and 0.82 Å for O—H, respectively. The *U*_iso_(H) = *xU*_eq_(C), where *x* = 1.2 for aromatic H.

### Crystal data

**Table:**

C_6_H_4_ClNO_3_	*Z* = 4
*M**_r_* = 173.55	*F*(000) = 352
Triclinic, *P*1	*D*_x_ = 1.622 Mg m^−^^3^
Hall symbol: -P 1	Mo *K*α radiation, λ = 0.71073 Å
*a* = 7.5390 (15) Å	Cell parameters from 25 reflections
*b* = 8.1640 (16) Å	θ = 9–13°
*c* = 13.132 (3) Å	µ = 0.49 mm^−^^1^
α = 94.75 (3)°	*T* = 293 K
β = 96.48 (3)°	Block, colourless
γ = 116.46 (3)°	0.20 × 0.10 × 0.10 mm
*V* = 710.9 (2) Å^3^	

### Data collection

**Table:**

Enraf–Nonius CAD-4 diffractometer	1833 reflections with *I* > 2σ(*I*)
Radiation source: fine-focus sealed tube	*R*_int_ = 0.075
Graphite monochromator	θ_max_ = 25.4°, θ_min_ = 1.6°
ω/2θ scans	*h* = 0→9
Absorption correction: ψ scan (North *et al.*, 1968)	*k* = −9→8
*T*_min_ = 0.909, *T*_max_ = 0.953	*l* = −15→15
2808 measured reflections	3 standard reflections every 200 reflections
2596 independent reflections	intensity decay: 1%

### Refinement

**Table:**

Refinement on *F*^2^	Secondary atom site location: difference Fourier map
Least-squares matrix: full	Hydrogen site location: inferred from neighbouring sites
*R*[*F*^2^ > 2σ(*F*^2^)] = 0.058	H-atom parameters constrained
*wR*(*F*^2^) = 0.171	*w* = 1/[σ^2^(*F*_o_^2^) + (0.1*P*)^2^ + 0.250*P*] where *P* = (*F*_o_^2^ + 2*F*_c_^2^)/3
*S* = 1.01	(Δ/σ)_max_ < 0.001
2596 reflections	Δρ_max_ = 0.32 e Å^−^^3^
200 parameters	Δρ_min_ = −0.36 e Å^−^^3^
0 restraints	Extinction correction: *SHELXL97* (Sheldrick, 2008), Fc^*^=kFc[1+0.001xFc^2^λ^3^/sin(2θ)]^-1/4^
Primary atom site location: structure-invariant direct methods	Extinction coefficient: 0.45 (3)

### Special details

**Table:**

Geometry. All e.s.d.'s (except the e.s.d. in the dihedral angle between two l.s. planes) are estimated using the full covariance matrix. The cell e.s.d.'s are taken into account individually in the estimation of e.s.d.'s in distances, angles and torsion angles; correlations between e.s.d.'s in cell parameters are only used when they are defined by crystal symmetry. An approximate (isotropic) treatment of cell e.s.d.'s is used for estimating e.s.d.'s involving l.s. planes.
Refinement. Refinement of *F*^2^ against ALL reflections. The weighted *R*-factor *wR* and goodness of fit *S* are based on *F*^2^, conventional *R*-factors *R* are based on *F*, with *F* set to zero for negative *F*^2^. The threshold expression of *F*^2^ > σ(*F*^2^) is used only for calculating *R*-factors(gt) *etc*. and is not relevant to the choice of reflections for refinement. *R*-factors based on *F*^2^ are statistically about twice as large as those based on *F*, and *R*- factors based on ALL data will be even larger.

### Fractional atomic coordinates and isotropic or equivalent isotropic displacement parameters (Å^2^)

**Table:**

	*x*	*y*	*z*	*U*_iso_*/*U*_eq_	
Cl1	0.09701 (18)	−0.12155 (17)	0.84727 (9)	0.0829 (5)	
O1	0.9021 (5)	0.6279 (4)	1.0905 (2)	0.0826 (10)	
O2	0.7781 (5)	0.5201 (4)	1.2241 (2)	0.0782 (9)	
O3	0.4570 (5)	0.2037 (4)	1.2061 (2)	0.0767 (9)	
H3A	0.5526	0.2962	1.2398	0.115*	
N1	0.7735 (5)	0.5097 (4)	1.1288 (3)	0.0597 (8)	
C1	0.4447 (6)	0.2008 (6)	0.8902 (3)	0.0629 (11)	
H1A	0.4394	0.1959	0.8188	0.075*	
C2	0.5999 (6)	0.3446 (5)	0.9564 (3)	0.0583 (10)	
H2A	0.7001	0.4381	0.9299	0.070*	
C3	0.6081 (5)	0.3513 (5)	1.0626 (3)	0.0489 (8)	
C4	0.4593 (6)	0.2115 (5)	1.1042 (3)	0.0539 (9)	
C5	0.3021 (6)	0.0657 (5)	1.0360 (3)	0.0580 (10)	
H5A	0.2021	−0.0294	1.0617	0.070*	
C6	0.2954 (6)	0.0625 (5)	0.9309 (3)	0.0590 (10)	
Cl2	0.29044 (16)	0.23975 (12)	0.58973 (8)	0.0630 (4)	
O4	0.1840 (5)	−0.5736 (4)	0.3805 (2)	0.0739 (9)	
O5	0.2094 (5)	−0.4362 (4)	0.2464 (2)	0.0739 (9)	
O6	0.2463 (5)	−0.3927 (4)	0.5626 (2)	0.0687 (8)	
H6A	0.2295	−0.4825	0.5222	0.103*	
N2	0.2030 (5)	−0.4369 (4)	0.3381 (2)	0.0540 (8)	
C7	0.2170 (5)	−0.1315 (5)	0.3516 (3)	0.0497 (9)	
H7A	0.2034	−0.1425	0.2798	0.060*	
C8	0.2359 (5)	0.0268 (5)	0.4076 (3)	0.0506 (9)	
H8A	0.2334	0.1225	0.3746	0.061*	
C9	0.2591 (5)	0.0401 (4)	0.5151 (3)	0.0460 (8)	
C10	0.2595 (5)	−0.1007 (5)	0.5650 (3)	0.0481 (8)	
H10A	0.2723	−0.0890	0.6368	0.058*	
C11	0.2407 (5)	−0.2611 (5)	0.5086 (3)	0.0474 (8)	
C12	0.2176 (5)	−0.2746 (4)	0.4005 (2)	0.0440 (8)	

### Atomic displacement parameters (Å^2^)

**Table:**

	*U*^11^	*U*^22^	*U*^33^	*U*^12^	*U*^13^	*U*^23^
Cl1	0.0728 (8)	0.0827 (8)	0.0728 (8)	0.0264 (6)	−0.0025 (6)	−0.0195 (6)
O1	0.085 (2)	0.0652 (19)	0.070 (2)	0.0088 (16)	0.0131 (16)	0.0168 (15)
O2	0.095 (2)	0.0759 (19)	0.0435 (17)	0.0233 (17)	0.0098 (14)	−0.0014 (13)
O3	0.093 (2)	0.0755 (19)	0.0431 (15)	0.0196 (16)	0.0213 (14)	0.0132 (13)
N1	0.070 (2)	0.0529 (18)	0.054 (2)	0.0260 (17)	0.0112 (16)	0.0083 (15)
C1	0.082 (3)	0.068 (3)	0.038 (2)	0.033 (2)	0.0116 (18)	0.0072 (18)
C2	0.070 (2)	0.058 (2)	0.046 (2)	0.027 (2)	0.0173 (18)	0.0112 (17)
C3	0.060 (2)	0.0470 (19)	0.0414 (18)	0.0262 (17)	0.0073 (16)	0.0053 (15)
C4	0.066 (2)	0.059 (2)	0.0397 (19)	0.0295 (19)	0.0159 (16)	0.0077 (16)
C5	0.063 (2)	0.053 (2)	0.057 (2)	0.0231 (19)	0.0211 (18)	0.0118 (17)
C6	0.063 (2)	0.059 (2)	0.056 (2)	0.032 (2)	0.0067 (18)	−0.0028 (18)
Cl2	0.0752 (7)	0.0489 (6)	0.0650 (7)	0.0330 (5)	0.0030 (5)	−0.0050 (4)
O4	0.114 (3)	0.0442 (15)	0.0637 (18)	0.0359 (16)	0.0214 (16)	0.0060 (13)
O5	0.099 (2)	0.0729 (19)	0.0451 (16)	0.0352 (17)	0.0187 (14)	−0.0026 (13)
O6	0.113 (2)	0.0499 (15)	0.0509 (16)	0.0420 (16)	0.0173 (15)	0.0176 (12)
N2	0.0596 (19)	0.0457 (17)	0.0481 (18)	0.0176 (14)	0.0108 (14)	−0.0003 (13)
C7	0.054 (2)	0.051 (2)	0.0372 (18)	0.0180 (17)	0.0070 (15)	0.0076 (15)
C8	0.057 (2)	0.0414 (18)	0.052 (2)	0.0206 (16)	0.0084 (16)	0.0141 (15)
C9	0.0472 (19)	0.0419 (18)	0.0468 (19)	0.0198 (15)	0.0061 (15)	0.0022 (14)
C10	0.058 (2)	0.0481 (19)	0.0352 (17)	0.0223 (17)	0.0073 (15)	0.0050 (14)
C11	0.058 (2)	0.0421 (18)	0.0399 (18)	0.0186 (16)	0.0129 (15)	0.0115 (14)
C12	0.0454 (19)	0.0372 (17)	0.0412 (18)	0.0112 (14)	0.0101 (14)	0.0055 (14)

### Geometric parameters (Å, º)

**Table:**

Cl1—C6	1.748 (4)	Cl2—C9	1.736 (3)
O1—N1	1.217 (4)	O4—N2	1.247 (4)
O2—N1	1.242 (4)	O5—N2	1.210 (4)
O3—C4	1.347 (4)	O6—C11	1.349 (4)
O3—H3A	0.8200	O6—H6A	0.8200
N1—C3	1.454 (5)	N2—C12	1.452 (4)
C1—C2	1.371 (5)	C7—C8	1.373 (5)
C1—C6	1.389 (6)	C7—C12	1.380 (5)
C1—H1A	0.9300	C7—H7A	0.9300
C2—C3	1.385 (5)	C8—C9	1.392 (5)
C2—H2A	0.9300	C8—H8A	0.9300
C3—C4	1.399 (5)	C9—C10	1.370 (5)
C4—C5	1.397 (6)	C10—C11	1.391 (5)
C5—C6	1.373 (5)	C10—H10A	0.9300
C5—H5A	0.9300	C11—C12	1.399 (5)
			
C4—O3—H3A	109.5	C11—O6—H6A	109.5
O1—N1—O2	121.8 (3)	O5—N2—O4	121.6 (3)
O1—N1—C3	120.0 (3)	O5—N2—C12	119.3 (3)
O2—N1—C3	118.2 (3)	O4—N2—C12	119.0 (3)
C2—C1—C6	119.2 (3)	C8—C7—C12	120.9 (3)
C2—C1—H1A	120.4	C8—C7—H7A	119.5
C6—C1—H1A	120.4	C12—C7—H7A	119.5
C1—C2—C3	120.3 (4)	C7—C8—C9	118.1 (3)
C1—C2—H2A	119.8	C7—C8—H8A	121.0
C3—C2—H2A	119.8	C9—C8—H8A	121.0
C2—C3—C4	120.7 (3)	C10—C9—C8	121.8 (3)
C2—C3—N1	117.8 (3)	C10—C9—Cl2	118.3 (3)
C4—C3—N1	121.5 (3)	C8—C9—Cl2	119.9 (3)
O3—C4—C5	116.6 (3)	C9—C10—C11	120.3 (3)
O3—C4—C3	125.0 (3)	C9—C10—H10A	119.9
C5—C4—C3	118.4 (3)	C11—C10—H10A	119.9
C6—C5—C4	120.0 (4)	O6—C11—C10	117.2 (3)
C6—C5—H5A	120.0	O6—C11—C12	124.8 (3)
C4—C5—H5A	120.0	C10—C11—C12	118.0 (3)
C5—C6—C1	121.3 (4)	C7—C12—C11	120.9 (3)
C5—C6—Cl1	119.1 (3)	C7—C12—N2	118.8 (3)
C1—C6—Cl1	119.7 (3)	C11—C12—N2	120.2 (3)
			
C6—C1—C2—C3	0.3 (6)	C12—C7—C8—C9	1.0 (5)
C1—C2—C3—C4	0.3 (6)	C7—C8—C9—C10	−1.2 (5)
C1—C2—C3—N1	−178.4 (4)	C7—C8—C9—Cl2	178.3 (3)
O1—N1—C3—C2	−2.3 (5)	C8—C9—C10—C11	1.4 (5)
O2—N1—C3—C2	177.5 (4)	Cl2—C9—C10—C11	−178.1 (3)
O1—N1—C3—C4	179.0 (4)	C9—C10—C11—O6	178.7 (3)
O2—N1—C3—C4	−1.2 (5)	C9—C10—C11—C12	−1.3 (5)
C2—C3—C4—O3	178.3 (4)	C8—C7—C12—C11	−0.9 (5)
N1—C3—C4—O3	−3.0 (6)	C8—C7—C12—N2	−178.2 (3)
C2—C3—C4—C5	−0.2 (5)	O6—C11—C12—C7	−178.9 (3)
N1—C3—C4—C5	178.5 (3)	C10—C11—C12—C7	1.0 (5)
O3—C4—C5—C6	−179.2 (4)	O6—C11—C12—N2	−1.6 (5)
C3—C4—C5—C6	−0.5 (6)	C10—C11—C12—N2	178.3 (3)
C4—C5—C6—C1	1.2 (6)	O5—N2—C12—C7	6.9 (5)
C4—C5—C6—Cl1	−180.0 (3)	O4—N2—C12—C7	−173.2 (3)
C2—C1—C6—C5	−1.1 (6)	O5—N2—C12—C11	−170.4 (3)
C2—C1—C6—Cl1	−179.9 (3)	O4—N2—C12—C11	9.5 (5)

### Hydrogen-bond geometry (Å, º)

**Table:**

*D*—H···*A*	*D*—H	H···*A*	*D*···*A*	*D*—H···*A*
O3—H3*A*···O2	0.82	1.91	2.605 (5)	142
O6—H6*A*···O4	0.82	1.88	2.581 (4)	143
O3—H3*A*···O6^i^	0.82	2.71	3.350 (5)	136
O6—H6*A*···Cl2^ii^	0.82	2.70	3.207 (3)	121
C2—H2*A*···O5^iii^	0.93	2.49	3.155 (5)	129

Symmetry codes: (i) −*x*+1, −*y*, −*z*+2; (ii) *x*, *y*−1, *z*; (iii) −*x*+1, −*y*, −*z*+1.
